# A novel method for standardized application of fungal spore coatings for mosquito exposure bioassays

**DOI:** 10.1186/1475-2875-9-27

**Published:** 2010-01-20

**Authors:** Marit Farenhorst, Bart GJ Knols

**Affiliations:** 1 Laboratory of Entomology, Wageningen University and Research Centre, PO Box 8031, 6700 EH, Wageningen, The Netherlands; 2 Div Infectious Diseases, Tropical Medicine & AIDS, Academic Medical Center, F4-217 Meibergdreef 9, 1105 AZ Amsterdam, The Netherlands

## Abstract

**Background:**

Interest in the use of fungal entomopathogens against malaria vectors is growing. Fungal spores infect insects via the cuticle and can be applied directly on the insect to evaluate infectivity. For flying insects such as mosquitoes, however, application of fungal suspensions on resting surfaces is more realistic and representative of field settings. For this type of exposure, it is essential to apply specific amounts of fungal spores homogeneously over a surface for testing the effects of fungal dose and exposure time. Contemporary methods such as spraying or brushing spore suspensions onto substrates do not produce the uniformity and consistency that standardized laboratory assays require. Two novel fungus application methods using equipment developed in the paint industry are presented and compared.

**Methods:**

Wired, stainless steel K-bars were tested and optimized for coating fungal spore suspensions onto paper substrates. Different solvents and substrates were evaluated. Two types of coating techniques were compared, i.e. manual and automated coating. A standardized bioassay set-up was designed for testing coated spores against malaria mosquitoes.

**Results:**

K-bar coating provided consistent applications of spore layers onto paper substrates. Viscous Ondina oil formulations were not suitable and significantly reduced spore infectivity. Evaporative Shellsol T solvent dried quickly and resulted in high spore infectivity to mosquitoes. Smooth proofing papers were the most effective substrate and showed higher infectivity than cardboard substrates. Manually and mechanically applied spore coatings showed similar and reproducible effects on mosquito survival. The standardized mosquito exposure bioassay was effective and consistent in measuring effects of fungal dose and exposure time.

**Conclusions:**

K-bar coating is a simple and consistent method for applying fungal spore suspensions onto paper substrates and can produce coating layers with accurate effective spore concentrations. The mosquito bioassay was suitable for evaluating fungal infectivity and virulence, allowing optimizations of spore dose and exposure time. Use of this standardized application method will help achieve reliable results that are exchangeable between different laboratories.

## Background

The rapid spread of insecticide resistance in disease vectors [1,2] has led to a renewed interest in biological control alternatives. Fungal entomopathogens can infect and kill many insect species and have been successfully used in agriculture [3-5]. More recent is the development of fungus-based methods for the control of malaria mosquitoes. The fungi *Metarhizium anisopliae *and *Beauveria bassiana *have been shown to effectively infect *Anopheles *mosquitoes and significantly reduce their lifespan [6-8]. Prior to death, fungal infection can decrease the mosquito's malaria transmission potential by reducing its feeding propensity, fecundity [9] and *Plasmodium *sporozoite levels [6]. Fungal entomopathogens are also effective against insecticide-resistant mosquitoes and increase their susceptibility to insecticides [10]. The potential of fungi to kill anophelines and reduce malaria transmission [11] has resulted in a growing interest to develop practical and sustainable mosquito control methods based on these biological control agents that can be integrated into the existing arsenal of malaria control tools [12,13].

Conidia of the hyphomycetous fungi *M. anisopliae *and *B. bassiana *are small (2-6 μm diameter), hydrophobic spores that can infect insects upon contact with the cuticle. Electrostatic and hydrophobic interactions allow conidia to attach to the insect's epicuticle [14,15] and subsequently germinate when humidity and nutrient conditions are conducive [16,17]. The production of germ tubes enables spores to penetrate the cuticle via mechanical pressure and cuticle-degrading enzymes [18]. After penetration, the fungus grows in the host haemocoel, taking up nutrients, destroying host cells and eventually killing the insect [19].

There are many different methods for infecting target insects with fungal spores. Dry conidia have been shown to be very effective in infecting mosquitoes in the laboratory [7]. However, dry fungal spores become air-borne when handled, which makes determinations of exposure dose inaccurate. The use of fungal suspensions allows for accurate quantifications of spore concentration with microscopy counts and is considered to be more feasible for large-scale experiments and field implementation.

Formulation can be an important determinant of spore infectivity and persistence [20]. Solvents that are suitable for applying hydrophobic conidia include Tween-water mixtures [21,22], solvents such as kerosene [23] or hydrocarbon isoparaffins (Shellsol T) [24], besides several oil-based solvents such as vegetable [25] and mineral oils (Ondina) [24,26,27]. The choice of solvent will depend on properties such as colour, odour and viscosity but also on the application method and substrate, which can affect the accessibility of spores to the insect after application. In general, oil formulations are considered to be beneficial for spore persistence in field situations as they can protect spores from desiccation [24,28,29].

For flightless insects, laboratory evaluations of dose and exposure time can make use of direct application of spore suspension on the insect cuticle [30,31]. Precise topical applications (with a pipette) are, however, not applicable for flying insects without using sedation, which can have a negative effect on their fitness and survival [32]. Topical applications are also less feasible for smaller sized insects. Mosquitoes can acquire spores via tarsal contact and obtain a lethal infection when resting on a fungus-impregnated surface [6,8,27]. It is thus not solely the spore concentration within the suspension but also the end concentration of spores per unit surface area that will determine the exposure dose.

There is currently no conventional application method for testing fungal spores against mosquitoes. To date, research on fungus formulations against mosquitoes has made use of brushing formulations on cotton cloths [8], dipping, i.e. submerging netting in fungus suspensions, manual spray applications on various substrates [33,34] and automated spray application inside clay pots [27]. Though all effective, none are very accurate in determining the effective end-concentration of fungal spores. Spraying is considered one of the more feasible application methods for larger scale experiments, but the loss of spores through "bounce-off" and run-off effects and the non-homogeneous spread of the spray make this method less accurate. The effective end-concentration of spores when sprayed onto paper was shown to be only around 10% of the estimated application dose [35]. To test effects of fungal dose and exposure time accurately, it is important to be able to apply specific amounts of fungal spores per unit surface area in a uniform and reproducible manner. The development of a standardized laboratory assay for testing fungal spores against mosquitoes, therefore, requires a novel and precise application method.

The paint and coatings industry has developed standardized and high precision methods for applying coatings onto substrates. Wired, stainless steel K-bars are designed with specifically sized grooves that coat solutions in a uniform layer of equal thickness. Here, the use of K-bars for applying fungus formulations on paper substrates was tested for use in mosquito bioassays. Effects of formulation and substrate on the infectivity of *M. anisopliae *and *B. bassiana *spores against *Anopheles gambiae s.s*. mosquitoes were tested and optimized using two novel application methods.

## Methods

### Fungus

Conidia of *Metarhizium anisopliae var. anisopliae*, isolate ICIPE-30 (courtesy Dr. N. Maniania, ICIPE, Kenya) and *Beauveria bassiana *Vuillemin isolate IMI 391510 were produced through solid state fermentation in aerated packed bed systems using glucose-impregnated hemp as a substrate (courtesy F. van Breukelen and M. Jumbe, Wageningen University, The Netherlands). After harvest, conidia were dried at ambient temperature until moisture content was < 5%. Prior to use, dry conidia were stored in 50 ml blue cap tubes in the dark at 4°C. *Beauveria *spores are white, round and on average measure a diameter of 2-4 μm. *Metarhizium *spores are green, have an elongated shape and on average measure a diameter of 4-6 μm.

For exposure time experiments (see below), a different production batch of *Beauveria bassiana *IMI 391510 spores was used. Conidia were produced at the laboratory of PennState University, USA on autoclaved barely flakes in mushroom spawn bags (courtesy Dr. N. Jenkins).

### Formulation

The most optimal formulation for the coating application method was empirically tested. The highly refined mineral oil Ondina 917 (Shell Ondina^® ^Oil 917, Shell, The Netherlands) and the synthetic isoparaffinic hydrocarbon solvent Shellsol T (Shell Shellsol T^®^, Shell, The Netherlands) have in previous studies shown to be useful as spore solvents, and were compared for their suitability in K-bar coating, separately, and in a 1:1 mixture. Spore solutions were homogenized through sonication at 1000 Hz for 10-15 seconds (Branson sonifier B12, Germany). Conidial concentrations were determined with a Bürker-Türk haemocyte counter (W. Schreck, Hofheim/TS) using a light microscope at a magnification of 400 × to quantify the amount of spores per ml. Conidial viability was assessed on Sabouraud dextrose agar enriched with 0.001% Benomyl, a fungicide that inhibits hyphal growth without affecting the germination, to facilitate spore counting [36]. Plates were kept in an incubator at 27°C for 22-26 hours. The proportion of germinated conidia was determined using a light microscope at magnification 400 ×. Stocks showing 85% or higher sporulation were used for experiments.

### Substrate

Two different substrates were tested: smooth, gloss-coated proofing paper that was provided with the K-bars consisting of wood-free Highland chromo paper 5415 (RK Print Coat Instruments Ltd., UK), and cardboard paper from file-folders made of 270 gram chlorine- and acid-fee Colorkraft cardboard (Jalema BV, Reuver, The Netherlands). Total volume and application methods were optimized for both paper types.

### Coater

**Figure 1:**
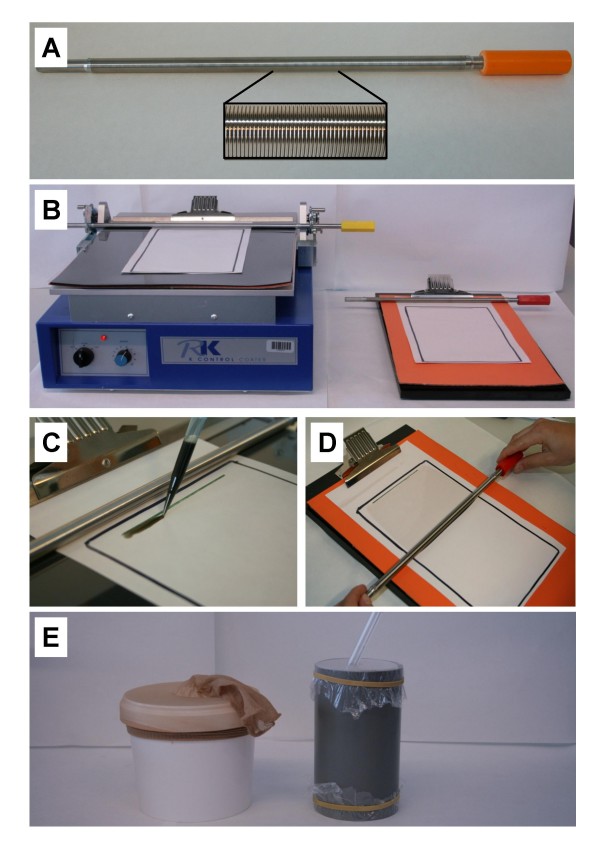
**K bar coating of fungal spores**. (A) The stainless steel K-bar with wired grooves (inset) that control film thickness. (B) Automated (left) and manual K-Coater (right). (C) Application of fungal spore suspension on the centre top part of the paper substrate with a pipette. (D) Coating a paper substrate with spore suspension using a swift top-to-bottom movement of the bar using light pressure. (E) The mosquito exposure set-up with a coated paper inside a PVC tube (right), sealed with plastic foil. Mosquitoes were transferred into the holding tube with an aspirator and (after exposure) to the holding buckets (left) via free flight.

Spore suspensions were applied onto substrates using wired K-bars (K bars^®^, RK Print Coat Instruments Ltd., United Kingdom), which were made of stainless steel rods with identically shaped grooves that control wet film thickness (Figure 1A). Two close wound K-bars were tested, with grooves of 0.15 or 0.31 mm that produced a coating thickness of 12 μm or 24 μm respectively.

The K-Hand Coater and the K-Control Coater Model 202 (RK Print Coat Instruments Ltd., UK) were compared (Figure 1B). The Hand Coater comprised a surface area of 220 × 340 mm and the Control Coater 250 × 325 mm and a soft coating bed consisting of three layers (a Melinex, foam and rubber layer on top of each other). Spore suspensions were applied manually onto the paper substrate with a pipette (Figure 1C). Using the Hand Coater, film deposits were applied manually by pulling the K-bar over the substrate in one rapid, smooth movement (Figure 1D). The K-Control Coater provided motorized applications that exerted a constant pressure between the K-bar and substrate and moved the bar over the substrate at a controlled speed. For experiments, application speed was maintained at 20 cm/sec. K-bars were wedged into the holder of the Control Coater and weights were adjusted for each bar to optimize pressure and horizontal position (using a water level). Bar settings and application speed were kept constant between replicates. Between application runs, spore residues were removed and killed by cleaning the K-bar and rubber bed with soft tissue paper drenched in 70% ethanol.

### Mosquitoes

Coatings were tested on laboratory-reared *An. gambiae s.s*. mosquitoes, originating from Suakoko, Liberia (courtesy of Prof. M. Coluzzi) and maintained in the Wageningen laboratory since 1989. Larvae were reared in plastic larval trays of 10 × 25 × 8 cm, filled with 1 L tap water at densities of approximately 0.3 larvae/cm^2^. Larvae were fed on Tetramin^® ^fish food (Tetra, Melle, Germany) daily, using 0.1 mg/larva for the first instars and 0.3 mg/larva for the other three larval stages. Pupae were collected daily and transferred to holding cages of 30 × 30 × 30 cm in which adults were maintained in climate controlled rooms (27 ± 1°C, 80 ± 10% RH and a 12 hr L:D photoperiod) and fed *ad libitum *on a 6% (w/v) glucose/water solution. For experiments, 3-6 days old females were used, which were selected using a mouth aspirator. When using the aspirator, no more than 20 females were aspirated into the tube at any given time.

### Bioassays

Coated papers were left to dry overnight in a climate room (27 ± 1°C, 70 ± 10% RH) before being placed inside a PVC-tube of 15 cm long and 8 cm diameter (Figure 1E). Papers covered the entire inside surface of the tube and were fixed with two small paperclips. Each tube was sealed with plastic microwave foil on either end, on which mosquitoes did not tend to rest. Mosquitoes were released in the tube via an aspirator (Figure 1E) and exposed for a fixed time period. In the first experiment, an exposure time of 6 hours was used, but an exposure time of 3 hours gave similar results and was therefore chosen for all other experiments. After exposure, mosquitoes were transferred to holding buckets of 20 cm diameter and 25 cm height, sealed with sheer nylon socks with the toe part cut off (Figure 1E), which during mosquito transfer were used to enfold one side of the PVC tube to facilitate mosquitoes flying into the holding bucket. Mosquitoes that died within 24 hrs were removed and survivors kept in a climate controlled room (27 ± 1°C, 80 ± 10% RH, 12 hr L:D photoperiod). Mortality was monitored daily, after which dead mosquitoes were removed from each bucket and checked for fungal infection by dipping cadavers in 70% ethanol and incubation on moist filter paper in sealed Petri dishes at 27 ± 1°C. After 3-5 days mosquito cadavers were examined for fungal sporulation, i.e., emerging hyphae, using a dissection microscope. Replicates were performed on separate days with fresh batches of mosquitoes.

### Experiments

#### Formulation experiments

Cardboard surfaces (15 × 25 cm) were coated manually with three different formulations of *M. anisopliae *or *B. bassiana *spores. One ml of Ondina suspension (10^10 ^spores/ml) was applied with a single movement of the 12 μm K-bar. Due to the lower viscosity and subsequent higher absorbance, 2 ml of 5 × 10^9 ^spores/ml was applied for the Shellsol and Shellsol/Ondina formulations using two bar movements (from top to centre and from bottom to centre) to reach the same end-concentration of 3 × 10^11 ^spores/m^2^. Control papers were treated with the same volumes of solvents without fungal spores. Per treatment, one group of 50 female *An. gambiae s.s*. mosquitoes was exposed for 6 hrs.

#### Substrate experiments

On proofing papers, 1 ml of a 10^10 ^spores/ml Shellsol suspension was applied with a single movement of the 12 μm K-bar. Cardboard papers were coated with 2 ml of 5 × 10^9 ^spores/ml to reach the same end-concentration of 3 × 10^11 ^spores/m^2^. For both paper types, three replicates of 50 female mosquitoes were exposed for 3 hrs to *Metarhizium*-coated, *Beauveria-*coated, or control papers (coated with 1 ml Shellsol).

#### Coater type experiments

Proofing papers were coated with 0.9 ml of 3.4 × 10^9 ^*B. bassiana *spores/ml Shellsol (= 10^11 ^spores/m^2^), using the 24 μm K-bar on the K Hand Coater or the K Control Coater. Control papers were treated with 0.9 ml Shellsol. Three replicate groups of 40 female mosquitoes were exposed to each treatment for 3 hrs.

#### Dose-response experiments

Proofing papers were manually coated, using the 24 μm K-bar, with 10-fold dilutions (taking 1 ml and adding 9 ml Shellsol) of the same stock suspensions of *M. anisopliae *and *B. bassiana*, resulting in end concentrations of 10^9^, 10^10^, 10^11 ^and 10^12 ^viable spores/m^2^. Control papers were treated with 0.9 ml Shellsol. Per treatment, one group of 40 female mosquitoes was exposed for three hours.

#### Exposure time experiments

Proofing papers were mechanically coated, using the 24 μm K-bar, with 0.9 ml of a suspension containing 4.2 × 10^9 ^or 4.2 × 10^10 ^*B. bassiana *spores/ml to reach end concentrations of 10^11 ^or 10^12 ^spores/m^2 ^respectively. Control papers were treated with 0.9 ml Shellsol. Per treatment, three replicate groups of 40 female mosquitoes were exposed for 5 min, 0.5 hr or 3 hrs.

### Data analysis

Effects of fungal infection on mosquito longevity were depicted in survival curves showing the cumulative daily proportional mosquito survival. Differences in mosquito survival between treatment and control groups were analysed using Cox Regression (P < 0.05) in SPSS 16.0 software. Cox Regression compared survival curves of different treatment groups [37], and gave significant differences in overall mortality rates in Hazard Ratio (HR) values, which indicate the average daily risk of dying. An HR value of 1 indicates equal mortality rates of both tested groups, a HR < 1 significantly lower overall mortality rates in group 2 compared with group 1, and *vice versa *for a HR > 1. For all data, Hazard Ratios were checked to be proportional over time graphically with plots of survivor functions to ensure the proportional hazard assumption was justified.

## Results

### Coating method

Coating applications were optimized for use in mosquito exposure tubes with a surface of 15 × 25 cm. This 0.0375 m^2 ^treatment surface was drawn onto an A4 size paper, which was attached onto the rubber K-coater holding board using the holding clasp (Figure 1B). Homogeneously mixed fungal suspensions were applied with a pipette in the centre of the top part of the treatment surface, not touching the bar prior to pulling (Figure 1C), to prevent fluid from spreading out of the surface boundaries. Manual pipetting required some practice to optimize uniformity and speed, and it was important to pull the K-bar over the paper rapidly (< 5 sec) after applying the suspension to prevent absorbance. Substrates such as cotton cloth or netting were not suitable for K-bar application. Porous cloth absorbed fluids too quickly and meshed netting gave insufficient contact between the K-bar and the suspension.

Manual application using the K-Hand Coater required practice to optimize speed, pressure and constancy of the bar pulling movement. The use of both hands on either side of the K-bar and applying light pressure was the most suitable mode of application (Figure 1D). Standardizing the K-Control Coater was easier and required only small adjustments of the bar settings and machine speed, which could remain fixed during experiments. Weights on both sides of the bar holder could be adjusted to optimize pressure and levelness (Figure 1B). The motorized bar-movement was optimized for A4 size papers and maintained at 20 cm/sec for all experiments.

The two K-bars tested, coating a thickness of 12 μm or 24 μm, were found to be equally suitable for the tested solvents. Less closely wound bars with larger grooves (> 50 μm) were not suitable for spreading liquids homogeneously. Tests started with the use of the 12 μm k-bar, which was suitable for coating cardboard substrates. For experiments with the proofing paper substrate, the 24 μm K-bar was most suitable for applying the test volumes of the thin Shellsol formulations. Therefore, after the formulation and substrate experiments, the 24 μm K-bar was used for all other experiments and chosen as the gold standard. For each application, the effective end-concentration of spores per m^2 ^was calculated using the spore concentration of the formulation (viable spores/ml), the total volume applied and the substrate surface.

### Formulation

Both Ondina oil and Shellsol solvent were suitable for suspending spores. Spores in Ondina remained suspended homogeneously for relatively long time periods (>2 hours) after mixing. Concentrations higher than 10^10 ^spores/ml, however, were too viscous for accurate applications with a pipette. The less viscous Shellsol allowed for homogeneous mixing, but spores did not remain suspended for long (<10 min) and required to be repeatedly re-mixed prior to application. Shellsol did, however, maintain >10^11 ^spores/ml whilst still allowing the use of a pipette and was suitable for microscopic spore counts where Ondina oil reduced the light quality and focus of the microscope. Ondina could be applied in small volumes (0.9 ml for 0.00375 m^2^) on various paper substrates, whereas for Shellsol such volumes could only coat a gloss-coated proofing paper in a single application movement.

Coated substrates were dried before use in exposure bioassays. Odourless Ondina oil and 1:1 Shellsol/Ondina mixtures did not evaporate and lengthy periods of drying (>16 hrs) were required for spores to be infective to resting mosquitoes. Shellsol dried within 1 hr, but more time (> 5 hrs) was required to remove its strong odour that could knock down mosquitoes. Drying time was standardized for a minimum of 18 hrs.

**Figure 2:**
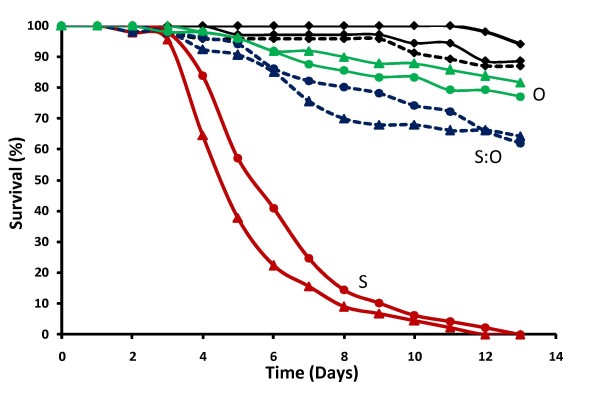
**Effect of formulation**. Cumulative daily proportional survival of female *An. gambiae s.s*. exposed for 6 hrs to cardboard papers manually coated with *M. anisopliae *spores (triangles) or *B. bassiana *spores (circles) formulated in Shellsol solvent (S, red), a 1:1 Shellsol/Ondina mix (S:O, blue) or Ondina oil (O, green). For each formulation, one group of 50 females was exposed to 3 × 10^11 ^viable spores/m^2^. Corresponding control groups (black) were exposed to the solvents only.

Infectivity of *M. anisopliae *and *B. bassiana *spores was tested when suspended in Ondina, Shellsol or 1:1 Shellsol/Ondina mixture and coated on cardboard papers with the K-Hand Coater. Mosquito survival data showed that Shellsol was the most effective solvent for both fungi, killing all mosquitoes within 13 days (Figure 2). Mosquito survival was significantly reduced compared with controls for both *M. anisopliae *(HR = 11.28, P < 0.001) and *B. bassiana *(HR = 10.02, P < 0.001), and respectively 91% and 93% of the mosquitoes showed fungal infection after death. The Shellsol/Ondina mix also reduced mosquito survival when applying *M. anisopliae *(HR = 3.89, P < 0.01) or *B. bassiana *(HR = 3.06 P < 0.01), though less than 40% of mosquitoes was killed within 13 days (Figure 2) and only 38% of the *Metarhizium*-exposed and 32% of the *Beauveria*-exposed showed infection after death. The Hazard Ratio values showed that fungi applied in a Shellsol/Ondina mix induced an approximate three times higher risk of dying in the infected mosquitoes, whereas using pure Shellsol formulation resulted in a fungus-induced risk of death approximately eleven times higher than the uninfected groups. Pure Ondina oil was the least effective formulation, giving no significant fungus-induced reductions in mosquito survival for *Metarhizium *(HR = 1.49; P = 0.18) or *Beauveria *(HR = 1.27, P = 0.31), and mosquito infection rates of only 12% and 18% respectively. Due to its short drying time and high infectivity, Shellsol was subsequently chosen as the standard coating formulation.

### Substrate

**Figure 3:**
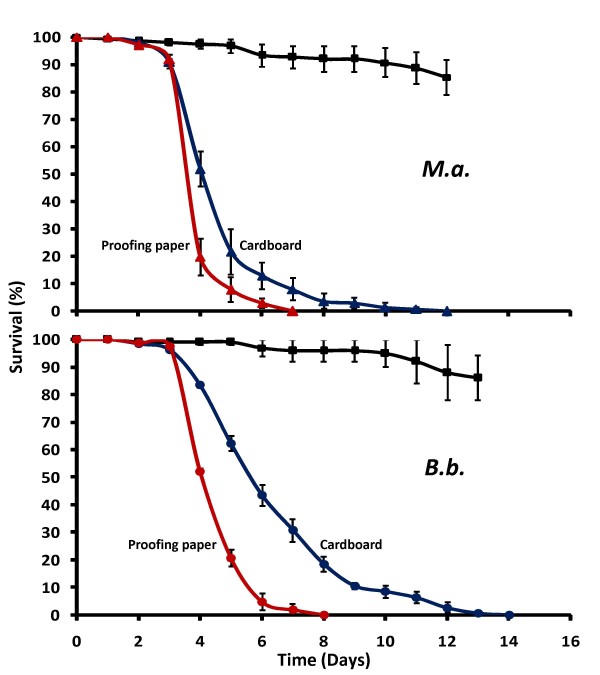
**Effect of substrate**. Cumulative daily proportional survival of female *An. gambiae s.s*. exposed for 3 hrs to proofing papers (red) or cardboard papers (blue) manually coated with 3 × 10^11 ^viable spores/m^2 ^Shellsol-formulated *M. anisopliae *(*M.a*.) or *B.bassiana *(*B.b*.). Controls were proofing papers coated with Shellsol only (black). Data represent the average ± SE survival of three replicates of 50 females.

The effect of substrate on spore infectivity was tested for smooth, gloss-coated proofing paper and more absorbent, thick cardboard papers. *Metarhizium anisopliae *significantly reduced mosquito survival compared to controls when applied on proofing paper (HR = 18.92, P < 0.001) or cardboard (HR = 15.65, P < 0.001) (Figure 3). *Beauveria bassiana *spores were equally infective, reducing mosquito survival when coated on proofing paper (HR = 17.67, P < 0.001) or cardboard (HR = 11.84, P < 0.001) (Figure 3). *Metarhizium*-coated proofing papers reduced mosquito survival more than coated cardboards (HR = 4.01, P < 0.001) and infected 92% of mosquitoes compared with 82% respectively. For *Beauveria *spores, differences in mortality rates were even larger between proofing papers and cardboard (HR = 5.12, P < 0.001), infecting 94% and 73% respectively. K-Coater proofing papers were, therefore, used as the standard coating substrate.

### Coater type

**Figure 4:**
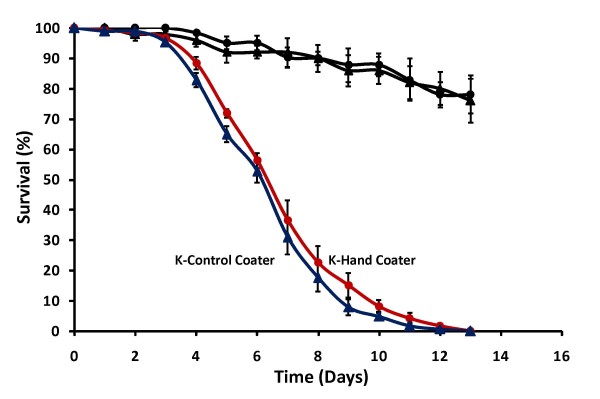
**Effect of coater type**. Cumulative daily proportional survival of *An. gambiae s.s *exposed for 3 hrs to controls (black) or 1 × 10^11 ^viable *B. bassiana *spores/m^2 ^coated manually with the K-Hand Coater (red) or mechanically with the K-Control Coater (blue). Data represent average ± SE survival of three replicates of 40 females.

The efficacy of manually applied spore coatings with the K-Hand Coater was compared with automated applications using the K-Control Coater. For both manually and mechanically applied *B. bassiana *coatings, significant reductions in mosquito survival compared to controls were obtained (HR = 15.31, P < 0.001 and HR = 14.84, P < 0.001 respectively) (Figure 4). Results were equally consistent and reproducible for both methods and the impact on mosquito survival was not significantly different (HR = 0.97, P = 0.9).

### Dose and exposure bioassays

**Figure 5:**
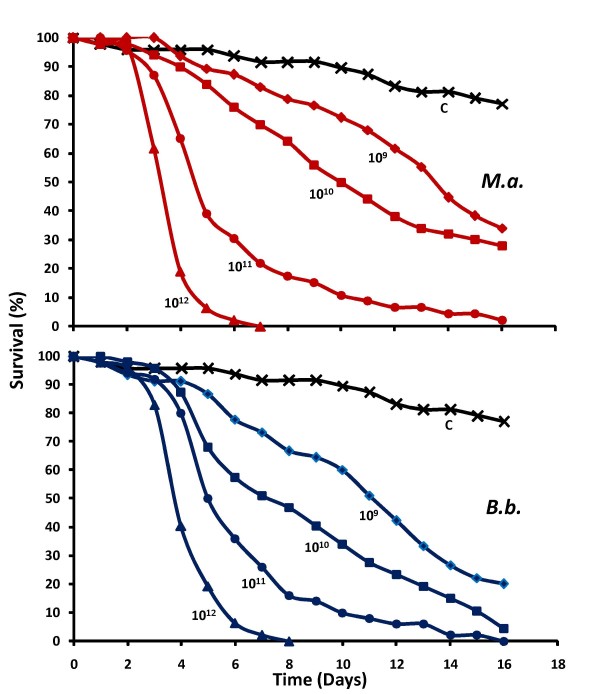
**Dose response curves**. Cumulative daily proportional survival of 40 female *An. gambiae s.s*. exposed for 3 hrs to control papers (black) or proofing papers manually coated with 10^9^-10^12 ^spores/m^2 ^of *M. anisopliae *(red) or *B. bassiana *(blue).

Dose-response curves were obtained for both *M. anisopliae *and *B. bassiana *by coating 10-fold dilutions of the same stock suspensions with the K-Hand Coater, resulting in end-concentrations ranging between 10^9 ^and 10^12 ^viable spores/m^2^. For both fungi, all tested doses reduced survival significantly compared with control mosquitoes (P < 0.001) (Figure 5). Mosquito survival data show a consistent dose-dependent increase in fungal virulence, with 10^9 ^spores/m^2 ^causing the smallest reduction in mosquito survival and 10^12 ^spores/m^2 ^the largest (Figure 5). Infectivity data also showed a dose-dependent increase for fungal infection. For *Metarhizium*, 19, 37, 76 and 95% of the mosquitoes showed fungal infection after death when exposed to respectively 10^9^, 10^10^, 10^11 ^and 10^12 ^spores/m^2^. For *Beauveria*, this was 23, 49, 84 and 90%.

**Figure 6:**
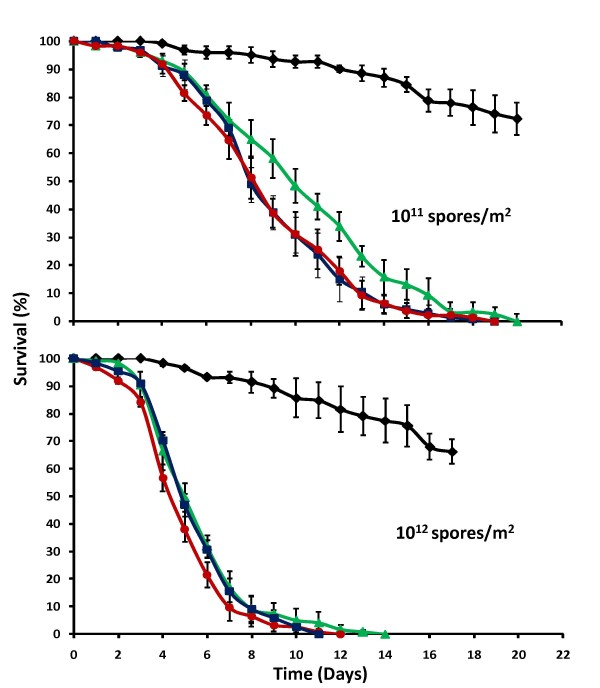
**Effect of exposure time**. Cumulative daily proportional survival of female *An. gambiae s.s*. exposed to control papers (black) or proofing papers mechanically coated with 10^11 ^or 10^12 ^*B. bassiana *spores/m^2 ^that were produced in PennState, USA. Data show survival curves (average ± SE) of three replicates of 40 females exposed for 5 min (green), 0.5 hr (blue) or 3 hrs (red).

The effect of mosquito exposure time on fungal infectivity and virulence was also tested. Three different exposure times (5 min, 0.5 hr and 3 hrs) were tested for two *B. bassiana *concentrations (10^11 ^and 10^12 ^spores/m^2^). As expected, the lower dose induced smaller reductions in survival than the higher dose (Figure 6). Interestingly, exposure time did not cause large differences in fungal virulence. Exposure for only 5 min was sufficient for reducing mosquito survival for both tested concentrations (P < 0.001). Only when exposed to the lower spore concentration did the 0.5 hr and 3 hrs exposures induce a significantly stronger reduction in mosquito survival (HR = 1.36, P = 0.016). For the higher dose, all exposure times resulted in similar reductions in mosquito survival (P > 0.05). When comparing the survival curves of the 3 hrs exposure time with those of the dose-response experiments for 10^11 ^and 10^12 ^spores/m^2^, the *Beauveria *spores produced using a bag-system in the USA (Figure 6) showed to be less virulent than the *Beauveria *spores produced by solid state fermentation in the Netherlands (Figure 5).

## Discussion

**Figure 7:**
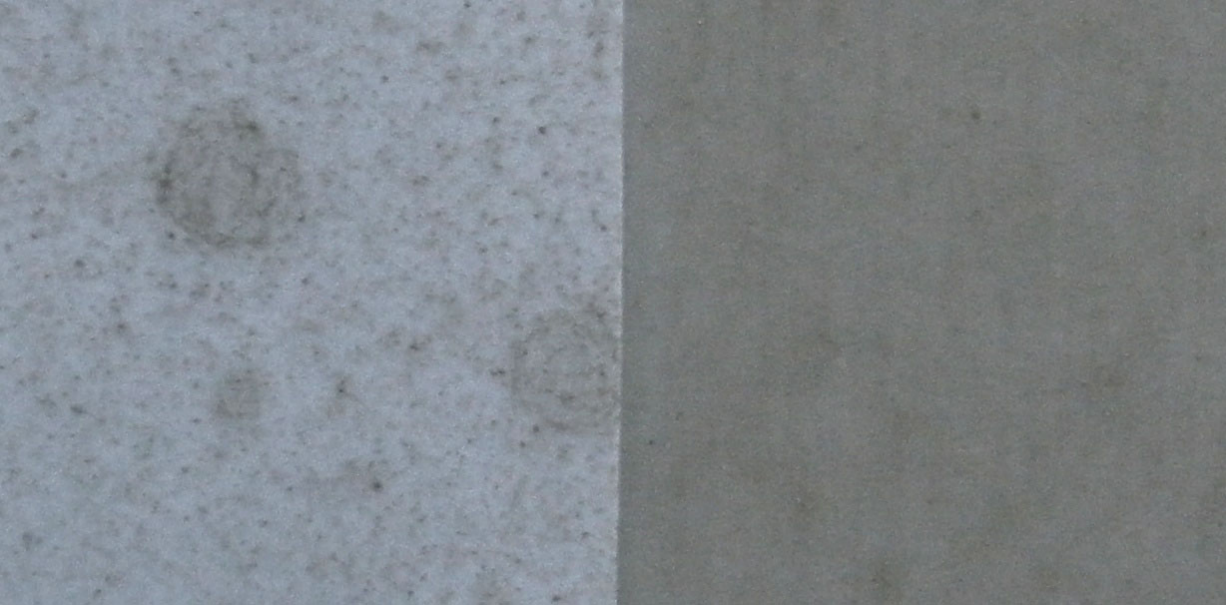
**Spore distribution**. Photo of a piece of proofing paper surface sprayed (left) or coated (right) with fungus formulation (zoomed in 4 ×). Equal volumes of a 5 × 10^9 ^spores/ml formulation (20% Ondina/80% Shellsol) were applied per surface area using the spray method described by Bell *et al *[35] or the optimized coating method. Spots on the left piece represent the spray droplets. The white background is not visible on the coated piece (right) because it is covered fully with the spore layer.

K-bar coating provided a simple and consistent method for coating layers of fungal spores onto paper substrates. By applying exact suspension volumes of known concentration onto a pre-determined substrate surface, the effective end-concentration of spores per unit surface area could be determined. The precision of the coating method could be somewhat affected by variations due to the manual use of the pipette and small proportions of formulation being coated outside the treatment surface boundaries or remaining on the K-bar as residue. These variations are, however, considered negligent compared to spraying, which has been reported to lose more than 90% of the application volume due to vaporization and bounce-off [35]. When applying the same volume per surface area, much more spores ended up on a coated paper compared with a sprayed paper, which is illustrated by its darker colour in Figure 7. The homogeneity of spore layers after application could not be quantified. Though fluorescent dyes may be used to improve visualisation of suspended spores [38], it was now not possible to quantify the number of spores with a microscope after application onto the paper. Therefore, the uniformity of spore coatings could only be determined visually. When using high *Metarhizium *concentrations, the K-bar deposited relatively homogeneous, non-clumping layers, where spraying would result in a more patchy distribution (Figure 7). The use of novel techniques such as quantitative PCR [35] may be used to quantify the spore layer of a coated paper and to determine the application efficacy and homogeneity of the coating method more precisely and allow more direct comparisons with spraying.

Viscosity of the solvent showed to be an important determinant of fungal infectivity and virulence (Figure 2). Though oil formulations have shown to be effective when sprayed on crops [39] and on porous materials such as clay pots [27], viscous Ondina oil was not a suitable solvent for spore coatings on papers (Figure 2). Ondina remained in the papers for several weeks, which may have caused strong adherence to the fungal spores that reduced their ability to attach to resting mosquitoes. The evaporative Shellsol solvent dried rapidly and kept spores adhered to the substrate whilst allowing attachment to mosquitoes. The type of substrate was also an important determinant of fungal infectivity (Figure 3). Gloss-coated proofing paper was the most effective substrate for Shellsol-formulated spores. Coatings on cardboard were less infective and virulent, especially for the smaller *Beauveria *spores (Figure 3). The higher porosity may have caused the spores to end up between the cardboard fibres instead of on the surface, causing them to be less accessible to mosquitoes. The optimal K-bar coating was a thin layer (12-24 μm) of Shellsol-formulated spores on a proofing paper substrate. Even though other Shellsol/Ondina mixtures may be found to be suitable as well, the aim of the formulation experiments was to find an effective solvent for coating applications, which in this case was pure Shellsol. The relatively short drying time of Shellsol allowed papers to be used shortly after application (≥ 5 hrs). Such a short timeframe between application and exposure is considered favourable for fungus-based mosquito bioassays to limit effects of potential reductions in spore viability over time.

The K-Hand Coater and the K-Control Coater were equally effective and consistent application methods for fungal spore coatings (Figure 4). The Control Coater allowed bar settings and applicator movement to be automated and kept constant between replicates. Between-user differences were not evaluated in this study, but it may be assumed that manual applications will be less consistent than automated applications. The K-Hand Coater, on the other hand, does not require electricity and has a smaller size and lower price, which makes it more appropriate for use in field laboratories.

Experiments on spore concentrations showed consistent dose-dependent effects of both *M. anisoplae *and *B. bassiana*. The speed of kill and mosquito numbers showing infection after death increased with increasing fungal dose. Even though only one replicate was tested per dose, consistent responses were obtained for both fungi. When using spraying as application method, applying higher doses does not always give a consistent increase in virulence (unpubl. data). Exposure time experiments showed that mosquitoes can pick up a lethal dose of fungal spores within a short period of five minutes. Differences in virulence were only observed when testing lower spore concentrations, indicating dose-dependent effects of exposure time.

K-bar coating was effective in applying both *M. anisopliae *and *B. bassiana *spores and did not require specific adjustments for each fungus. The coating method may also be optimized for other fungus species, target insects and other types of exposure assays. Variables, including bar type, substrate and formulation can be varied to achieve the most appropriate method for customized bioassays.

The aim of this study was to obtain a simple, precise and consistent mode of application of fungal spores for use in laboratory assays. Other application methods may prove to be more effective in terms of spore infectivity or more feasible for field application. For instance, spraying of fungal spores seems to require fewer spores for obtaining similar infectivity and virulence to mosquitoes compared to our coated papers [35]. For field implementation, spraying may likely also prove more effective and feasible than coating. An optimal field delivery method has other requirements than a laboratory method, such as high spore persistence. The most feasible and sustainable method for applying fungi in field settings needs to be determined and effects of formulation, substrate and dose measured under field conditions. For laboratory applications, however, the most important requirements are high precision and repeatability. Though spraying has been shown to be very effective, it results in a large loss of spores (> 90% [35]) and a patchy distribution (Figure 7). Spore clumps may result in effective infections but will not allow for adequate exposure dose estimations. With K-bar coating, a more precise volume of spore formulation is applied on the treatment surface in an even layer thickness with one single movement, resulting in a more uniform spore spread (Figure 7) and much less contamination of the work space. By providing more precise estimates of the effective fungal exposure dose, the coating method may be a valuable tool for laboratory testing of lethal and sub-lethal effects of fungal infection on malaria mosquitoes. Testing spores on Shellsol-coated papers can also be useful for laboratory persistence assays and screening for the most persistent fungal isolate

Since many institutes are collaborating in the development of a fungus-based malaria vector control tool, the use of a single, standard fungus-mosquito bioassay will be a valuable improvement. There are currently no standard conventional methods for fungus application and mosquito exposures. Mosquito survival results of studies using different application and exposure methods cannot be directly compared because of the differences in the effective exposure dose. Effects of other parameters, such as mosquito species, formulation or spore production methods on fungal infectivity and virulence can only be tested when using a single application method. For instance, we observed differences in virulence of the *Beauveria *spores produced by Wageningen (Figure 5) and PennState (Figure 6), which can be further explored, also between laboratories, only when a single application and exposure method is chosen. The use of an existing, purchasable applicator will allow for easy standardisations of the K-bar coating method between institutes. This novel method may provide the application method for standard fungus-mosquito bioassays that are crucial for achieving appropriate and exchangeable results between laboratories.

## Conclusions

• K-bar coating provided a simple, effective and consistent application method for fungal spores. Variables can be adjusted for customized fungus-insect bioassays.

• Formulation and substrates were important determinants of fungal infectivity. Low viscosity Shellsol formulations and smooth, non-absorbent proofing papers were most suitable for coating.

• Manually (K-Hand Coater) and mechanically (K-Control Coater) applied fungal spore coatings showed similar and reproducible effects on mosquito survival.

• With high spore concentrations (≥ 10^11 ^spores/m^2^), mosquito exposure times as short as 5 minutes were sufficient to induce lethal fungal infections.

• Virulence increased step-wise with increasing fungal dose and can, with this application method, be optimized for different mosquito experimental settings.

## Competing interests

The authors declare that they have no competing interests.

## Authors' contributions

MF designed the study, performed the experiments and statistical analyses, and drafted the manuscript. BGJK contributed to the study design and writing of the manuscript. Both authors read and approved the final manuscript.
